# Editorial: Occupational health and organizational culture within a healthcare setting: challenges, complexities, and dynamics

**DOI:** 10.3389/fpubh.2023.1327489

**Published:** 2023-11-21

**Authors:** Yvonne Tran, Louise A. Ellis, Robyn Clay-Williams, Kate Churruca, Siri Wiig

**Affiliations:** ^1^Centre for Healthcare Resilience and Implementation Science, Australian Institute of Health Innovation, Macquarie University, Sydney, NSW, Australia; ^2^SHARE – Centre for Resilience in Healthcare, Faculty of Health Sciences, University of Stavanger, Stavanger, Norway

**Keywords:** occupational health, organizational culture, healthcare, wellbeing, healthcare professional (HCP), safety culture, staff wellbeing, burnout

The ever-changing healthcare demands and the challenges posed by global health crises have prompted the healthcare sector to give increased importance to occupational health. Stemming from the realization that a productive and sustainable workforce is rooted in the health and wellbeing of workers, there is growing interest in formulating and implementing strategies that help identify, prevent, and manage occupational health risks (1). This trend is expected to continue as employers aim to enhance the health and safety of their employees while improving organizational culture, performance, and competitiveness (2–4). Institutional priorities have shifted significantly to focus on providing sufficient support to healthcare workers in these areas. Recent papers, some of which are featured in this Research Topic, explore various facets of this topic, such as the nuanced interplay of labels and concepts in healthcare settings and how to approach learning when the system fails and patients are harmed (Wiig et al.). The research reported in this Research Topic features the critical role of professional engagement, the challenges and solutions surrounding workplace violence, and the need for continuous training in fields like nursing (Al-Mugheed et al.; Wang, Tang et al.; Yu et al.). Moreover, these studies emphasize the importance of adaptability and resilience in healthcare systems and teams (Fagerdal et al.).

The wellbeing of healthcare professionals, encompassing their physical, mental, and emotional health, is deeply intertwined with the quality of care delivered to patients (5). Organizational culture plays a pivotal role in this dynamic, setting the tone for how healthcare professionals engage with their work, colleagues, and patients. A positive and supportive organizational culture fosters collaboration, continuous learning, and resilience, directly contributing to enhanced patient outcomes and professional satisfaction. Conversely, a negative culture can lead to burnout, reduced efficiency, and compromised patient care (3). As such, understanding and cultivating a healthy organizational culture is critical for the holistic wellbeing of healthcare professionals and the patients they treat (6). Examining these issues, several papers on the Research Topic offer valuable insights into the influence of organizational culture on healthcare providers and their patients. For instance, the study by Babaie et al. in neonatal intensive care units explores the multifaceted nature of safety culture from the perspective of frontline medical staff in Iran. Ellis, Tran et al. showed the adaptability and resilience of healthcare systems during challenging organizational changes, emphasizing the role of a supportive workplace culture in their empirical study of an Australian hospital undergoing significant transformation. The poignant commentary from Montgomery and Lainidi, highlights the urgent need for systemic shifts in healthcare organizational cultures, a sentiment further amplified by the global challenges presented by the COVID-19 pandemic. In a different cultural setting, Wang, Zhang et al. developed a scale for the hospital organizational environment in China, reflecting its vital role for understanding the values and behaviors of both clinicians and nursing staff. As the world continues to grapple with the COVID-19 crisis, the case study by Paquay et al. recounts the benefits of post-shift clinical debriefings, signifying the integral role of organizational strategy in ensuring patient safety and bolstering clinician wellbeing. Lastly, the salient perspective article by Ellis, Falkland et al. critically reflects the intricacies and challenges tied to defining, measuring, and improving safety culture in healthcare. Understanding organizational culture is crucial, as it directly influences the mental wellbeing of its workers, shaping workforce burnout and staff retention. In the aftermath of the COVID-19 pandemic, burnout is one of the most prevalent staff wellbeing problems, with the ability to being able to retain and attract healthcare workers being fundamental to the ability to provide healthcare services in the future.

An integral aspect of occupational health, especially in sectors like healthcare, is understanding and addressing professionals' mental and emotional wellbeing. The psychological state of health care workers can significantly affect their cognitive capacity, influencing their performance, decision-making, and interactions with others (7). The psychological wellbeing of healthcare professionals, including their emotional resilience and coping mechanisms, can also directly influence the quality of care they provide to patients. This mental and emotional health is affected by the demands of their profession and work environments. Examining these concerns, several papers within this Research Topic shed light on the intricate relationship between the psychological health of healthcare workers and the environments they navigate. For instance, Seaward et al. highlight the occupational health and safety issues within residential aged care, suggesting potential neglect of worker wellbeing. Arad et al. investigated the interplay between teamwork and the psychological safety of surgical staff in Israel. Similarly, a study conducted in Germany by Treusch et al. examined the association between job satisfaction and the mental health of physician assistants, which encompassed facets such as general job satisfaction, work-related factors, and mental health indicators. This study paints a comprehensive picture of the challenges experienced. Yang et al. explored job burnout among primary health workers in China, illuminating the protective role of work-family support. The demanding nature of anesthesia work and its implications for staff wellbeing is captured in the study from Khalafi et al. The detrimental effects of incivility in hospitals, as discussed by Pavithra et al., emphasize the significance of a respectful and nurturing workplace culture for maintaining staff wellbeing. Together, these papers demonstrate the profound impact of workplace environments on the psychological health of healthcare professionals and, consequently, the care and care quality they deliver to patients.

In the complex and dynamic landscape of healthcare, the importance of the wellbeing of healthcare workers stands out more than ever. Occupational health plays a significant role in determining the quality and efficiency of patient care. As global health challenges continue to emerge, healthcare professionals' resilience, adaptability, and mental fortitude are tested, underscoring the need for supportive and nurturing workplace cultures. The research presented in this Research Topic serves as a testament to the impacts on safety and wellbeing of the intricate relationship between organizational culture, workplace environment, and the health of healthcare professionals. It reinforces the idea that a positive organizational culture uplifts the spirits of those working within its confines, directly translating to better patient outcomes. As we reflect on these findings, several questions arise: How can healthcare institutions uphold and enhance positive organizational cultures amidst resource constraints and mounting external pressures? What measures can be taken to bridge the gap between recognizing the importance of occupational health and implementing effective solutions? How can interventions be tailored to address the unique challenges diverse healthcare settings face worldwide? And given the ongoing impact of the COVID-19 pandemic, how can healthcare systems better equip themselves-both in terms of infrastructure and the wellbeing of their healthcare workers-to respond to future global health crises? The insights gleaned from this Research Topic show a pressing need to prioritize and invest in the wellbeing of healthcare professionals. As the backbone of the healthcare industry, their physical and psychological health inevitably determines the quality of care patients receive. Moving forward, it is imperative for stakeholders at all levels, from policymakers to hospital administrators, to commit to fostering environments that champion the holistic wellbeing of healthcare professionals, thereby ensuring a brighter and healthier future for all.

## Author contributions

YT: Writing – original draft. LE: Writing – review & editing. RC-W: Writing – review & editing. KC: Writing – review & editing. SW: Writing – review & editing.
